# Impact of Periodontitis on Endothelial Risk Dysfunction and Oxidative Stress Improvement in Patients with Cardiovascular Disease

**DOI:** 10.3390/jcm13133781

**Published:** 2024-06-27

**Authors:** Angela Angjelova, Elena Jovanova, Alessandro Polizzi, Ludovica Laganà, Simona Santonocito, Rosalia Ragusa, Gaetano Isola

**Affiliations:** 1 University Dental Clinical Center St. Pantelejmon, Skopje, Faculty of Dentistry, Ss. Cyril and Methodius University in Skopje, 1000 Skopje, North Macedonia; 2 Department of General Surgery and Surgical-Medical Specialties, School of Dentistry, University of Catania, 95124 Catania, Italy; 3 Health Direction of Policlinic Hospital, 95100 Catania, Italy; ragusar@unict.it

**Keywords:** cardiovascular disease, periodontitis, endothelial dysfunction, periodontal treatment, stroke, atherosclerosis, arterial stiffness, oxidative stress

## Abstract

Periodontitis is a multifactorial chronic inflammatory disease that affects the periodontium and overall oral health and is primarily caused by a dysbiotic gingival biofilm, which includes, among others, Gram-negative bacteria such as *Porphyromonas gingivalis*, *Actinobacillus actinomycetemcomitans*, and *Tannerella forsythensis* that colonize gingival tissues and that can lead, if not properly treated, to periodontal tissue destruction and tooth loss. In the last few decades, several large-scale epidemiological studies have evidenced that mild and severe forms of periodontitis are strictly bilaterally associated with several cardiovascular diseases (CVDs), stroke, and endothelial dysfunction. Specifically, it is hypothesized that patients with severe periodontitis would have compromised endothelial function, a crucial step in the pathophysiology of atherosclerosis and several CVD forms. In this regard, it was postulated that periodontal treatment would ameliorate endothelial dysfunction, hence bolstering the notion that therapeutic approaches targeted at diminishing cardiovascular risk factors and different forms of periodontal treatment could improve several CVD biomarker outcomes in the short- and long-term in CVD patients. The aim of this review is to update and analyze the link between periodontitis and CVD, focusing on the inflammatory nature of periodontitis and its correlation with CVD, the effects of periodontal therapy on endothelial dysfunction and oxidative stress, and the impact of such therapy on CVD biomarkers and outcomes. The article also discusses future research directions in this field.

## 1. Introduction

Periodontal diseases (PDs) represent significant health challenges and pathological conditions affecting the periodontium, terminology denoting the supportive structure encompassing the tooth. This structure comprises the gingival tissue, alveolar bone, cementum, and periodontal ligament [1,2]. PDs signify a widespread universal health problem, with severe forms of periodontitis affecting approximately 9% of the population. It is recognized as the 10th most prevalent disease of all. The pathogenic development of PD is marked by intricate interactions among microorganisms within dental plaque and the host’s immune-inflammatory reaction. This reaction can be affected by genetic causes, environmental circumstances, and acquired conditions such as smoking and systemic diseases [3,4]. These diseases comprise cardiovascular diseases, gastrointestinal and colorectal cancer, diabetes, Alzheimer’s disease, autoimmune diseases, medications, respiratory infections, and pregnancy consequences. The existence of periopathogens and their metabolic by-products within the oral cavity may indeed influence the immune response outside the oral cavity, potentially fostering the emergence of systemic disorders [5,6].

A collective from the American Heart Association (AHA) has asserted that, despite the absence of conclusive evidence establishing a causative link between periodontitis and cardiovascular disease (CVD), a noticeable association still exists. The postulated mechanism underlying this association suggests that individuals with periodontitis may exhibit heightened vulnerability to the access of oral bacteria and bacterial constituents to the bloodstream, facilitated by an inflamed and ulcerated pocket epithelium [7]. Proposed mechanisms elucidating the association between PD and CVD involve shared inflammatory pathways, including elevated levels of white blood cells, C-reactive protein, fibrinogen, intracellular adhesion molecule-1 (ICAM-1), and proinflammatory cytokines. Moreover, both diseases exhibit overlapping risk factors, such as smoking, reduced oral hygiene, diabetes mellitus, obesity, and stress. However, it is challenging to conclude that periodontitis constitutes a primary causal factor of CVD due to the complexity of confounding factors that link PD to CVD and lower quality of life [8,9,10].

This review article is designed to provide current insights into the potential association that serves as the foundation for comprehending the involvement of PD in cardiovascular events. It aims to examine the evidence gathered from numerous scientific studies, elucidating the correlation. 

Furthermore, the article intends to explore the impact of diverse periodontal treatments on enhancing CVD outcomes and biomarkers. Finally, the article summarizes the current state of knowledge concerning the conceivable links between PD and its role as a risk factor in precipitating cardiovascular occurrences, covering developments over the past two decades. 

## 2. Materials and Methods

An electronic search was conducted on PubMed, Scopus, and Web of Science according to the following strategy: “Periodontal treatment AND (endothelial dysfunction OR oxidative stress)”. A total of 1497 records (801 from Pubmed, 45 from Scopus, and 651 from Web of Science) were screened for their titles and abstracts by two independent reviewers. Articles that did not meet the preliminary relevance criteria were excluded at this stage. Full texts of the remaining articles were retrieved and reviewed in detail to develop the topic. A hand search was also performed on Google Scholar to include all potential articles related to the aim of the present review. The following inclusion criteria were adopted: (1) studies published in peer-reviewed journals, (2) articles written in English, (3) full-text available, (4) study design (original articles and reviews), and (5) relevance to the topic (role of periodontitis and periodontal treatment in endothelial dysfunction and oxidative stress). Articles that were not peer-reviewed (e.g., editorials, commentaries, and letters), studies not written in English, and studies not directly related to the topic were excluded.

## 3. Periodontitis as an Inflammatory Disease and Its Correlation with Cardiovascular Disease

Individuals afflicted with periodontitis demonstrate inflammatory degradation of the supportive tissue of the tooth. The distinctive features include the depletion of connective tissue and collagen within the gingiva, with the loss of the periodontal ligament and the resorption of alveolar bone [11]. While the precise etiology of the onset and advancements of periodontal disease remains unidentified, scientific consensus acknowledges its complicated nature, stemming from various contributing elements. A crucial role in its pathogenesis is attributed to a disturbance in the oral microbiome, an intricate system that disrupts dental structures, thereby influencing a localized immune and inflammatory response [12]. Periodontitis commonly originates from the preceding gingivitis, although not all cases of gingivitis advance to periodontitis [13]. The shift in the composition and pathogenic potential of plaque microorganisms, in conjunction with host resistance factors and tissue-related mechanisms, acts as determining factors for the transition from gingivitis to periodontitis and the subsequent progression of periodontal destruction [14]. As a chronic inflammatory condition, periodontitis demonstrates a foundational immune dysregulation characterized by aberrant immune function. This involves the simultaneous interplay of multiple causal components [15].

The primary focus in the control of periodontal disease lies in the early and precise diagnosis of the condition, given that addressing extensive damage to the periodontal bone and soft tissue can be challenging. Diagnosis of periodontal diseases predominantly relies on radiographic and clinical evaluations of the periodontal tissues. By utilizing clinical parameters such as bleeding on probing, probing depth, and clinical attachment level, along with radiographic assessment of supporting bone, an accurate diagnosis can be established [16]. Effective management of these pathological conditions leads to a substantial enhancement in the general health status of the individual. The approach to treating periodontal disease is presently advancing owing to the acknowledgment of the specific plaque hypothesis of periodontal disease [17].

**Figure 1:**
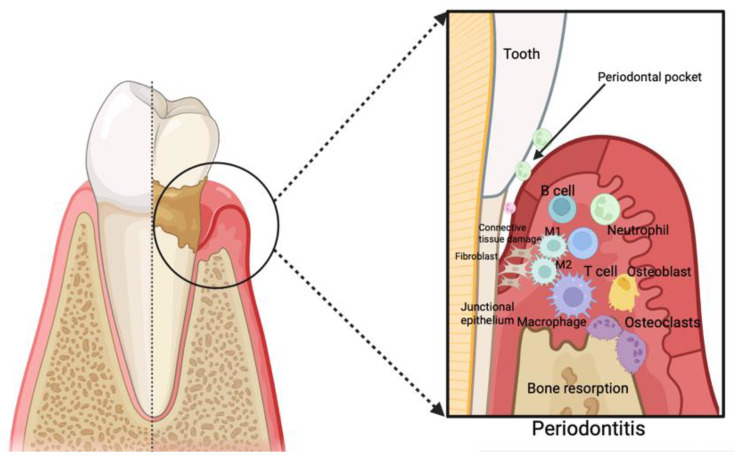
Immune response during periodontal disease. Created with BioRender.com (accessed on 6 June 2024).

Typically, the initiation of the inflammatory cascade of periodontitis involves the migration of phagocytes, specifically neutrophils and macrophages, to the site of the lesion. This progression is facilitated by the junctional epithelium, which liberates mediators such as interleukins (ILs), prostaglandin E2 (PGE2), and tumor necrosis factor-alpha (TNF-alpha) [18]. The phagocytic cells express specific receptors on their plasma membrane that identify and bind surface molecules of bacteria, such as Toll-like receptors (TLRs). The primary purpose involves the elimination of microorganisms, followed by the effective removal of cellular debris [19,20]. If microorganisms persist in their growth or if there is an impaired immune response, the acute phase of periodontal inflammation transitions into a chronic state, leading to the generation of additional mediators. These developments prompt the employment of diverse immune cells, including T-cells (Figure 1). Consequently, the inflammatory cascade induces bone resorption by osteoclasts, the degradation of ligament fibers by matrix metalloproteinases (MMPs), and the formation of granulation tissue [15,20,21].

Given that dental plaque is the primary etiological factor in the development of PD, the foremost strategy for preventing this condition involves effective oral hygiene practices and the removal of plaque. The mechanical manipulation, involving ultrasonic scaling, of both supra and subgingival surfaces of teeth with or without the incorporation of antimicrobial agents is designed to disrupt the biofilm and initiate the inflammatory response in the host [22,23]. The persistent and generally gradual development of this condition leads to tooth mobility, diminished chewing functions, aesthetic disturbances, and, if untreated, eventual tooth loss. Furthermore, periodontal inflammation has systemic repercussions, triggering low-grade systemic inflammation that adversely impacts other organs [11]. 

In recent years, multiple investigations have documented epidemiological links between periodontitis and CVD [24]. Cardiovascular diseases encompass a spectrum of heart and vascular disorders such as atherosclerosis, ischemia, peripheral artery disease, infective endocarditis, and acute cardiac infarction [25]. DNA damage has emerged as a significant risk factor contributing to the development of atherosclerosis and coronary artery disease. It arises from various endogenous and exogenous factors including oxidative stress, aging, smoking, hypertension, hyperlipidemia, and diabetes mellitus. Table 1 summarizes emerging risk factors for cardiovascular disease, which encompass multiple overlapping categories. Failure to repair such damage can result in mutations, potentially predisposing individuals to disease [26]. Research investigating inflammation and coronary artery disease (CAD) has revealed that proinflammatory cytokines possess the capability to enlist leukocytes, lymphocytes, and macrophages, thereby initiating a localized inflammatory reaction. This cascade prompts the proliferation of smooth muscle cells and the deposition of extracellular matrix, potentially accelerating atherosclerosis. Additionally, it can induce variability in the fibrous cap, predisposing it to plaque rupture and subsequent myocardial infarction in certain cases [27]. Due to their infectious and inflammatory nature, periodontal diseases are hypothesized to contribute to atherogenesis through mechanisms facilitated by oral microorganisms and the resultant inflammation. Microbes linked to periodontal diseases may inhabit atheromatous plaques and potentially exacerbate their degradation by activating local inflammatory responses, thus spreading the inflammatory cascade implicated in the formation, progression, and eventual rupture of atheroma [28].

Inflammatory mediators and markers are found at elevated levels in the systemic circulation of patients with periodontitis compared to periodontally healthy patients. There are two potential ways through which this could arise. Firstly, the mediators generated within periodontal lesions, including cytokines and others, may disseminate into the bloodstream, utilizing influences on tissues and organs away from the oral cavity. Consequently, this dissemination could induce inflammatory alterations in the endothelium, such as increased expression of adhesion molecules and the stimulation of cytokine synthesis. Secondly, microorganisms have the potential to trigger systemic inflammatory reactions by interacting with serum lipids, activating receptors present in inflammatory cells and endothelial cells, invading endothelial cells, or colonizing atheromatous lesions with bacterial or bacterial-derived components [29].

## 4. Impact of Periodontal Therapy on Endothelial Dysfunction in Individuals with Periodontitis

Vascular endothelial cells (ECs) form a continuous lining along the internal surfaces of the entire vascular network. This endothelial layer comprises approximately 6 × 10^13^ cells, with a total weight of 1.5 kg, covering an estimated surface area of around 1000 square meters within the human body. The physiological roles of ECs in normal conditions encompass the regulation of various physiological processes, such as responding to infections and sepsis, managing coagulation and fibrinolysis, controlling vessel diameter, and modulating blood flow [30]. 

In reaction to unfavorable stimuli, vascular endothelial cells (VECs) undergo a phenotypic alteration toward an activated state, commonly referred to as endothelial dysfunction (ED). This dysfunction has been evidenced to play a pivotal role in the initiation and progression of various vascular pathologies, including atherosclerosis, hypercholesterolemia, diabetes, and hypertension [31].

Endothelial dysfunction, a precursor to atherosclerosis, begins with the buildup of leukocytes on the vessel wall, eventually migrating into the subendothelial space. This progression is facilitated by cell adhesion molecules present in both white blood cells and endothelial cells [32]. While the exact mechanisms responsible for endothelial dysfunctions are complex and involve multiple factors, there is mounting evidence suggesting that the elevated production of reactive oxygen species (ROS) may play a significant role in the occurrence. Additionally, it signifies a disparity between the factors involved in vasoconstriction and vasodilatation. The endothelium-derived relaxing factor, recognized as nitric oxide (NO), possesses strong antiatherosclerotic properties. NO, released by endothelial cells, collaborates with prostacyclin to deter platelet aggregation, hinder the attachment of neutrophils to endothelial cells, and suppress the expression of adhesion molecules. Moreover, in elevated concentrations, NO impedes the proliferation of smooth muscle cells. Consequently, in circumstances where there is an absolute or relative deficiency of NO, the onset of progression of atherosclerosis is promoted [30,33]. 

**Figure 2:**
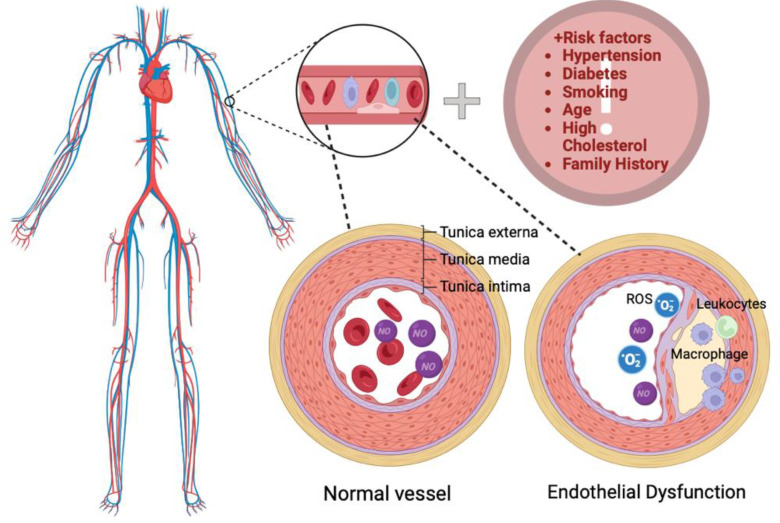
The underlying mechanisms of endothelial dysfunction resulting from diminished vascular NO bioavailability. Cardiovascular risk factors such as hypertension, diabetes mellitus, smoking, aging, menopause, and familial history of CVD. This process leads to the adhesion and infiltration of inflammatory cells like macrophages and neutrophils into the vascular wall, culminating in intimal proliferation. Created with BioRender.com (accessed on 6 June 2024).

Endothelial dysfunction (ED) and mild inflammation exhibit a close association. Chronic periodontitis induces a mild systemic inflammatory state. Persistent systemic inflammation resulting from periodontitis may lead to endothelial dysfunction through various mechanisms, either singularly or in combination. These mechanisms include reduced bioavailability or the production of NO, as well as increased inactivation of NO (Figure 2). ED exacerbates inflammation within the vascular wall, leading to the upregulation of adhesion molecules and cytokines [34]. The presence of periodontal pathogens and their harmful stimuli, along with periodontal cytokines, can be recognized by receptors present on VECs. This recognition triggers the initiation of inflammatory pathways. Among the specialized pattern-recognition receptors (PRRs), toll-like receptor-2 (TLR-2) and TLR-4 are the most studied, as they are pivotal in recognizing periodontal bacteria [35,36,37]. Multiple studies have demonstrated the presence of bacteria within atherosclerotic plaques. It is established that periodontal pathogens and their byproducts enter the bloodstream, correlating with endothelial dysfunction and the development of atherosclerotic lesions. These findings unequivocally indicate that oral microorganisms can infiltrate the walls of blood vessels. However, it remains uncertain whether they directly contribute to atherosclerosis or merely invade already damaged arteries [38]. Many biomarkers can be used to determine endothelial dysfunction, as shown in Table 2.

Three distinct hypotheses have been proposed to establish a connection between periodontitis and ED, namely the bacteriological hypothesis, the inflammatory hypothesis, and the immune hypothesis [30]. 

The bacteriological hypothesis involves *Porphyromonas gingivalis,* which plays a dual role not only in promoting inflammation and tissue degradation within periodontal diseases but also in contributing to the inflammatory processes affecting distant organs, including the development of atherosclerosis. It gains access to the bloodstream through epithelial ulcers following various treatment interventions. *P. gingivalis* and its components, such as fimbriae and DNA, have been identified within human atherosclerotic plaques. The invasive and persistent characteristics of *P. gingivalis* are pivotal for inducing ED, which in turn enhances the production of pro-inflammatory mediators and adhesion molecules, thereby increasing leukocyte adhesion to the vascular wall [30,39]. 

In the inflammatory hypothesis, the activation of endothelial cells results in heightened expression of selectins, vascular cell adhesion molecule-1 (VCAM-1), and intercellular adhesion molecule-1 (ICAM-1), facilitating the adherence of monocytes that transform into macrophages. Macrophages then transit into foam cells, prompting the synthesis of proinflammatory cytokines, thereby triggering endothelial dysfunction [30,40]. This theory posits that inflammatory mediators such as IL-1 and TNF-alpha enter the bloodstream, eliciting systemic inflammatory responses such as the production of acute-phase proteins [41].

The immune hypothesis postulates that host defenses provide protection, but aberrant immune reactions can lead to heightened tissue damage. This atypical inflammatory response, termed a “hyper-responsive” phenotype, is associated with various inflammatory conditions, characterized by an amplified inflammatory reaction upon Toll-like receptor (TLR) stimulation [42]. Individuals exhibiting this phenotype are at increased risk of developing periodontitis, with the overexpression of pro-inflammatory mediators exacerbating the risk of ED. An alternative immunological theory posits an autoimmune response directed at heat-shock proteins (HSP) [30]. The host immune system, sensitized by HSP from a predominant periodontal pathogen like *P. gingivalis*, is believed to exhibit cross-reactivity with the corresponding mammalian protein in the gingival connective tissue. This phenomenon is also implicated in the development of atherosclerosis [43].

Periodontal therapy exerts a beneficial influence on endothelial dysfunctions, a critical determinant of cardiovascular health. Interventions such as subgingival biofilm ultrasonic debridement have been shown in various studies to enhance vascular function and mitigate inflammatory markers. One study showed that following periodontal treatment, there was a notable enhancement in the dilation of the brachial artery, and it may be feasible to induce the regression of the atherosclerotic process during the stage of endothelial dysfunction through the treatment of chronic inflammation [44]. A study also demonstrated that addressing periodontal infection could potentially result in decreased levels of inflammatory markers and enhanced vascular endothelial function [45]. Additionally, other findings revealed that individuals afflicted with periodontitis exhibited severe endothelial dysfunction, a condition that notably improved after three months of conservative treatment, excluding surgical procedures [46]. Research demonstrates that immediately following intensive periodontal treatment, there may be temporary systemic inflammation and endothelial dysfunction. However, six months post-treatment, the clinical advantages of periodontal treatment are linked to a considerable enhancement in endothelial function [47].

## 5. Impact of Periodontal Therapy on Oxidative Stress in Patients Affected by Periodontitis

Reactive oxygen species (ROS) induce tissue injury through diverse mechanisms, encompassing DNA damage, lipid peroxidation (via the activation of cyclooxygenases and lipoxygenases), protein damage affecting components such as gingival hyaluronic acid and proteoglycans, and the oxidation of crucial enzymes such as antiproteases [48]. There has been increasing focus in recent years on reactive oxygen species (ROSs) due to their pivotal role in the advancement of numerous inflammatory conditions involving periodontitis. They play a role in regular cellular metabolic processes and are consistently produced by cells in the majority of tissues [49]. The comprehensive review of oxidative stress in chronic periodontitis has been extensively discussed in prior studies. Free radicals and ROSs play critical roles in numerous physiological functions. However, when the antioxidant system fails to neutralize their effects adequately, it can lead to tissue damage, exacerbating periodontal damage [50]. ROSs typically coexist within all aerobic cells in equilibrium with biochemical antioxidants. Recently, heightened oxidative stress and compromised antioxidant defenses have been proposed as potential factors contributing to the onset and advancement of complications in coronary artery diseases [26]

**Figure 3:**
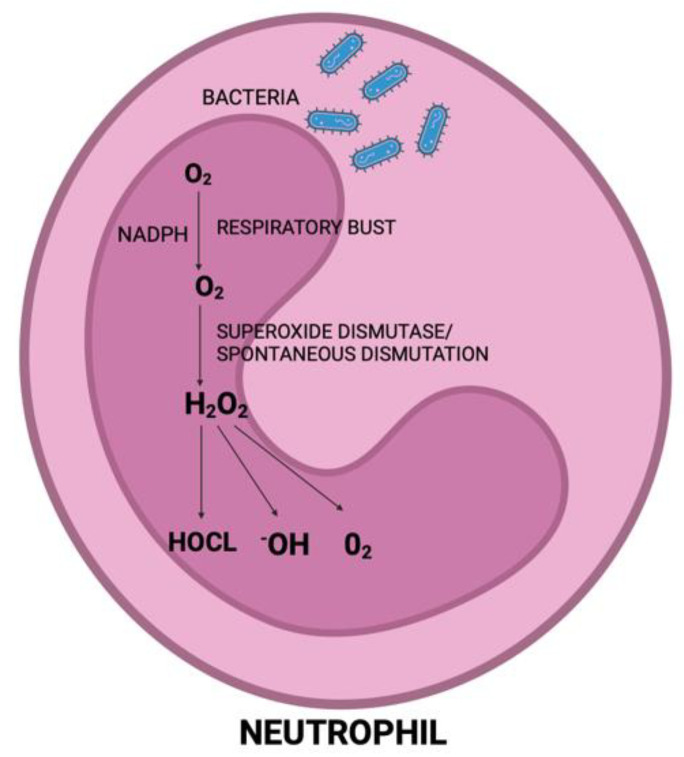
The generation of reactive oxygen species (ROSs) during periodontal disease occurs following the internalization of pathogens by neutrophils. This process leads to the production of superoxide anions. These anions can undergo enzymatic conversion via superoxide dismutase to yield hydrogen peroxide. Hydrogen peroxide, in turn, serves as a precursor for various derivates, including hypochlorous acid and singlet oxygen. Created with BioRender.com (accessed on 6 June 2024).

Neutrophils constitute the predominant population of leukocytes in the bloodstream and serve as the primary defense against bacterial infections. Following the activation of the host response by pathogenic biofilms, neutrophils are recruited as the predominant inflammatory cells, congregating within periodontal tissues and gingival sulcus. They are recognized as the primary contributors to ROS production in periodontitis [51]. During phagocytosis, free radicals, particularly O_2_, H_2_O_2_, and OH^−^, which arise as byproducts of the mitochondrial respiratory burst in polymorphonuclear neutrophils, primarily induce lipid peroxidation (Figure 3). Additionally, they can cause damage to proteins and DNA. This results in an oxidative imbalance that initiates proinflammatory pathways, crucially promoting osteoclastogenesis. Consequently, this process contributes to the bone loss observed in patients with periodontitis [52].

When homeostatic equilibrium is disrupted, attributed to an increased concentration of ROSs or a reduction in antioxidant capacity, oxidative stress emerges. This condition is associated with various systemic ailments, including cardiovascular disease, arthritis, and diabetes. Moreover, the scientific literature indicates the potential involvement of oxidative stress in the development of periodontal diseases, as heightened ROS production by inflammatory cells may contribute to tissue degradation [53].

### 5.1. Oxidative Stress Biomarkers

For many years, the physiological roles of free radicals have been overlooked, while considerably more attention has been directed towards understanding the pathological implications of oxidative stress. Various types of free radicals are generated with diverse substrates, resulting in a spectrum of biomarkers that offer the potential for evaluating oxidative stress-induced harm and could elucidate the inflammatory state of periodontal tissues. Therapeutic interventions might help reestablish their levels to those observed in individuals with healthy periodontal conditions [53,54].

For a biomarker to possess diagnostic validity, it must meet specific criteria. Primarily, it necessitates high sensitivity to accurately detect the presence of diseases. Secondly, the biomarker must exhibit the ability to differentiate between diseased individuals and healthy individuals with high specificity. Lastly, the biomarker should demonstrate precision and reproducibility in consistently characterizing the disease across the population [55].

Numerous biomarkers of oxidative damage have been identified, with the most prominent ones targeting lipid peroxidation, such as malondialdehyde (MDA), then oxidized LDL, MDA-modified LDL, and auto-antibodies against oxidized LDL [56]. The oxidative stress biomarkers associated specifically with oxidative damage included 8-hydroxydeoxyguanosine (8-OHdG), 4-hydroxynonenal (HNE), and myeloperoxidase (MPO). Furthermore, biomarkers specifically linked to antioxidant mechanisms included glutathione peroxidase (GSH-Px) and superoxide dismutase [53]. Well-established salivary indicators of oxidative stress encompass thiobarbituric acid-reacting substances (TBARSs) indicating lipid peroxidation, advanced oxidation protein products (AOPPs) denoting protein oxidation, and advanced glycation end products (AGEs) reflecting protein glycation and, consequently, carbonyl stress closely linked to oxidative stress. Total antioxidant capacity (TAC) or ferric-reducing antioxidant power (FRAP) can be quantitatively measured to evaluate antioxidant status. These markers are all detectable in saliva. Other oxidative stress biomarkers and antioxidants are summarized in Table 3. Despite notable biological variations influenced by an expanding array of external factors, their concentration in saliva offers valuable insights into the condition of oral tissues [57].

Oxidation damages both DNA and RNA in various disease conditions, including PD and CVD. Additionally, protein carbonyl (PC), recognized as a marker of protein oxidation, has been monitored in individuals with periodontitis and CVD. Moreover, the total antioxidant capacity (TAOC) serves as an encompassing parameter reflecting the collective activity of nonenzymatic antioxidants found in plasma and bodily fluids [53,55,58]. Individuals afflicted with periodontitis exhibited elevated levels of MDA and 8-OHdG in their saliva, gingival fluid, and blood compared to periodontally healthy individuals serving as controls [59].

#### 5.1.1. 8-Hydroxydeoxyguanosine (8-OHdG)

8-Hydroxydeoxyguanosine (8-OHdG) stands as the predominant constant outcome of oxidative DNA damage induced by reactive oxygen species (ROS). Multiple investigations have underscored the significance of 8-OHdG levels in bodily fluids as a biomarker of oxidative stress. It has been demonstrated to elevate the bodily fluids and tissues of individuals suffering from various conditions, including diabetes, cancer, atherosclerotic cardiovascular disease, rheumatoid arthritis, and more recently, chronic periodontitis (CP) [60]. Research findings indicated that salivary concentrations of 8-OHdG were elevated in individuals diagnosed with CP, and these levels demonstrated a reduction after initial periodontal therapy. Moreover, notably higher levels of 8-OHdG concentrations alongside diminished total antioxidant status were observed in the gingival blood of patient groups affected by periodontitis [61].

Another study documented a correlation between the levels of 8-OHdG and the abundance of *P. gingivalis* in saliva samples obtained from individuals with periodontitis [59]. The abundance of certain bacteria, including *P. gingivalis*, *Treponema denticola*, and *Tannerella forsythia*, significantly impacted the concentration of 8-OHdG. This association between the presence of periodontopathic bacteria and the biomarker highlights how the extent of inflammation affects the level of 8-OHdG in saliva [62].

#### 5.1.2. Malondialdehyde (MDA)

The extent of oxidative stress can be quantified by evaluating the production of malondialdehyde (MDA) levels, which serve as a terminal byproduct of lipid peroxidation (LPO). Elevated hydroxyl radical levels, an oxygen-derived free radical, result in excessive MDA production. Research findings from various studies indicate a notably higher concentration of salivary MDA in periodontitis compared to periodontally healthy tissues [48,63]. Existing research suggests that elevated levels of MDA are associated with various conditions such as cancer, atherosclerosis, and diabetes, along with a higher prevalence among smokers [64]. Elevated concentrations of MDA have been documented in inflamed periodontal tissue, suggesting potential involvement in the destructive mechanisms of periodontitis. This underscores the possible contribution of ROS to the pathogenesis of periodontal disease [65].

The elevation in salivary MDA levels may result from the generation of the superoxide anion (O_2_) during the interaction between periopathogens or their byproducts and neutrophils within periodontal tissues or pockets. Another investigation suggested that the compromised antioxidant defense system, caused by the excessive production of LPO products at sites of inflammation, could contribute to heightened oxidative stress in individuals with periodontitis [66].

### 5.2. Arterial Stiffness and Endothelial Dysfunction Outcomes

Arteries dilate as they receive blood propelled from the heart during systole, subsequently releasing it to the periphery during diastole to maintain a consistent blood flow throughout both cardiac cycles. Nonetheless, with aging and in various disease states, there is a reduction in the compliance and distensibility of arteries, leading to diminished elasticity in the vessel walls, a condition referred to as “arterial stiffness”, which serves as an indicator of these altered arterial properties [67]. Arterial stiffness is regarded as an indicator of arteriosclerosis and is posed as a predisposing factor for cardiovascular disorders [68].

Given the evidence linking chronic inflammation to the onset of coronary heart disease (CHD), a causal link between periodontal disease and CHD has been postulated. Consequently, considerable interest has been focused on investigating the independent association between periodontal disease and CHD [69]. Indeed, heightened arterial stiffness correlates with alterations in nitric oxide production, compromised endothelial function, and diminished vasodilation [68]. The primary vascular alterations associated with heightened arterial stiffness include vascular fibrosis resulting from collagen accumulation, the degradation of elastic fibers, the bonding of collagen and elastin fibers through advanced glycation end products, and significant calcification of vessel walls [67,70].

Numerous investigations have documented elevated arterial stiffness in individuals afflicted with periodontitis in contrast to control groups, indicating that those with periodontitis may experience subclinical vascular impairment [71,72,73].

Previous studies have utilized various markers linked to this condition, including carotid intima-media thickness (CIMT), flow-mediated vasodilatation (FMD), and the assessment of arterial stiffness, which can be also conducted non-invasively through the measurement of pulse-wave velocity (PWV) [74,75].

Pulse wave velocity (PWV) represents the rate at which pressure waves, initiated by cardiac contraction, flow through the arterial structure. The assessment of PWV yields insights into the elastic characteristics of the arterial system. Elevated PWV readings indicate reduced vessel compliance and, consequently, increased arterial rigidity. The preferred method for measuring PWV is through carotid-femoral assessment, considered the gold standard in clinical practice. Research indicates that arterial stiffness is influenced by inflammation and oxidative stress pathways, leading to an elevation in PWV concurrent with an increase in systemic inflammatory markers [76,77].

PWV is assessable across different segments of the vascular system, determined through applanation tonometry, which involves dividing the distance between two points within the vasculature by the time delay of the waveforms at these locations. PWV has been utilized to investigate arterial stiffness in periodontitis; however, the findings across studies vary due to inconsistencies in how periodontitis was evaluated. Addressing several limitations, a novel ultrasound-based technique known as Pulse Wave Imaging (PWI) was developed. PWI enables the direct visualization of vessel interiors and wave propagation, facilitating the estimation of regional PWV. Moreover, PWI offers both quantitative and qualitative insights into arterial health [78,79].

Some studies revealed that individuals suffering from periodontitis exhibited elevated arterial stiffness, as evidenced by higher PWV values when compared to those without periodontitis, and as a result, the authors concluded that periodontitis correlates with heightened arterial stiffness [73,75,80,81].

Flow-mediated vasodilation (FMD) serves as an indicator of endothelial function and is also a predictor of cardiovascular occurrences, regardless of conventional cardiovascular risk factors. The assessment of FMD in the brachial artery has been utilized to evaluate endothelial function in human subjects [82]. This method relies on the principle that heightened flow in the brachial artery induces shear forces, triggering the activation of endothelial cells. Consequently, endothelial cells produce nitric oxide (NO) through the action of nitric oxide synthase, ultimately resulting in vasodilation. In cases where vasodilation fails to achieve 5%, there is evident endothelial dysfunction [83] Moreover, a previous study demonstrated a notable reduction in FMD among patients diagnosed with periodontitis compared to control subjects [84].

Carotid intima-media thickness (CIMT) is a characteristic of arterial wall aging and correlates with subclinical atherosclerosis due to certain cellular and molecular alterations. It has been recognized as an indicator of arterial damage. A prior meta-analysis revealed that periodontal disease accompanied by elevated markers of systemic bacterial exposure was linked to a notable rise in CIMT compared to individuals without periodontitis, particularly among those with severe periodontitis [85]. Another study showed that a direct and dose-dependent correlation was observed among periodontal pocket depth, CIMPT, and arterial stiffness [71]. In individuals without functional atherosclerosis, CIMT demonstrates a positive correlation with tooth loss, while this association is not observed in those with functional atherosclerosis. These results contribute to understanding the impact of arterial stiffness progression on tooth loss [86].

Several studies have demonstrated that periodontal treatment significantly reduces oxidative stress. Elter suggests that enhancements in FMD could become evident within just a month following periodontal treatment, excluding the use of antibiotics [45]. Another study observed that following periodontal therapy, the levels of 8-OHdG in GCF and saliva significantly decreased in both groups of patients with periodontitis [87]. Moreover, an improvement in glycemic status was observed three months after scaling and root planning (SRP), likely due to the reduction in inflammatory and oxidative stress associated with the SRP procedure [88].

## 6. Impact of Periodontal Treatment on CVD Biomarkers and Outcomes

There is mounting interest in assessing the effects of periodontal treatment on early biomarkers associated with the risk of early CVD, as well as examining how biomarker levels influence the long-term effectiveness of periodontal therapy [89]. Randomized controlled trials (RCTs) have demonstrated that periodontal treatment effectively diminishes the presence of pathogenic microorganisms within dental plaque, as well as systemic levels of IL-6, C-reactive protein (CRP), and E-selectin [47,90,91]. Additionally, evidence from RCTs suggests that periodontal treatment enhances blood pressure, endothelial function, and the lipid profile [47,92,93]. Based on current data, it is recognized that untreated or poorly managed moderate to severe periodontitis raises systemic inflammation, which may independently heighten the risk of CVD [94]. One study showed that periodontal therapy affected endothelial-dependent function, as evidenced by a 2% absolute difference observed between the test and control groups six months after therapy [95]. Recent randomized controlled clinical trials have demonstrated enhancements in cardiovascular surrogate markers (such as endothelial function, sICAM, hsPCR level, and fibrinogen) following periodontal treatment. However, these trials are constrained by limitations regarding external validity, periodontal treatment strategies, and the consistency or extrapolation of results [96].

A preliminary investigation revealed that among individuals with periodontitis and no systemic illnesses, initial non-surgical periodontal therapy enhanced compromised endothelial function within 6 weeks post-treatment, without the use of antimicrobial agents. In two pilot studies, non-surgical periodontal therapy, which involved antibiotics, resulted in a notable enhancement of endothelial dysfunction among patients with severe periodontitis, observed three months following treatment [97]. The intensive periodontal therapy group, in one study, exhibited a decline in FMD 24 h after treatment but experienced a significant improvement in FMD at 2 and 6 months compared to the control treatment group. The amelioration of ED was associated with reductions in the number of periodontal pockets and bleeding on probing [97,98].

Another study showed that no substantial variance in brachial CIMT was observed before and three months following non-surgical periodontal treatment incorporating systemic antimicrobial therapy. However, in more recent studies, a decrease in CIMT was documented subsequent to periodontal treatment [99]. Certain findings indicated a notable reduction in CIMT at 6- and 12-months post-treatment. The decrease in CIMT was observed at various locations along the carotid axis, including the carotid bifurcation. These observations suggest a favorable impact of periodontal treatment on CIMT [97]. Earlier research demonstrated that intensive periodontal treatment leads to a temporary elevation in inflammatory marker levels in the bloodstream and exacerbates endothelial function, potentially due to the release of bacteria or inflammatory cytokines into circulation. However, after several weeks, both local and systemic inflammatory markers, as well as periodontal disease parameters, decreased, resulting in significantly improved endothelial function compared to pre-treatment levels. Additionally, there is a reduction in CIMT observed six months following periodontal treatment [8].

## 7. Future Research on Periodontal Treatment and Cardiovascular Health

Future research on exploring the impact of periodontal treatment on ED and oxidative stress in patients with CVD should delve into several promising areas. Studies with larger, more diverse populations could provide a deeper understanding of the causal relationship and long-term benefits of periodontal therapy on cardiovascular health. Advanced molecular techniques could elucidate the specific biochemical pathways through which periodontal therapy mitigates oxidative stress and improves endothelial function. Additionally, investigating the interplay between genetic predisposition and the efficacy of periodontal interventions could pave the way for personalized treatment approaches. Integrating metagenomic analyses and exploring microbiota–host interactions will be pivotal in developing targeted interventions that not only treat periodontal disease but also mitigate their impact on systemic conditions [100]. The future of dental practice could undergo significant changes if further research confirms that periodontal disease is a genuine risk factor for atherosclerosis-related conditions and that periodontal treatment can prevent or slow the development of these medical issues.

## 8. Conclusions

Available evidence suggests that periodontal therapy effectively improves established CVD risk factors and serves as an effective strategy for ED management. The primary outcome following periodontal treatment was a decrease in serum levels of CRP, indicative of systemic inflammation stabilization, alongside enhancements in ED measures, representing markers of CVD. Based on the reviewed evidence, periodontal therapy demonstrates beneficial effects on blood inflammatory markers and improves the lipid profile. Considering the established correlation between periodontal disease and CVDs, collaboration between dentists and cardiologists is advocated to promote primary prevention and offer personalized therapy for both periodontal and cardiopathic patients.

## Figures and Tables

**Table 1:** Novel prospective risk indicators for cardiovascular disease: C-reactive protein (CRP), cardiovascular diseases (CVDs), flow-mediated dilation (FMD), interleukins (ILs), and low-density lipoprotein (LDL).

Inflammatory markers	Fibrinogen, plasma viscosity, CRP, IL-6, TNF-alpha, MMP-8, MMP-9, MMP-13, antibodies, and low-density lipoprotein
Markers of endothelial dysfunction	Oxidized low-density lipoprotein, brachial artery reactivity, glutathione dysfunction
Thrombotic markers	Tissue plasminogen activator, homocysteine, von Willebrand factor
Non-invasive imaging biomarkers	Ankle brachial pressure index, magnetic resonance imaging angiography, carotid intima-media thickness, CT coronary calcification
Invasive imaging biomarkers	Intravascular ultrasound, coronary angiography
Oxidative stress biomarkers	Acrolein, malonyldialdehyde (MDA), 4-hydroxynonenal (HNE), 8-hydroxydeoxyguanosine (8-OHdG), protein carbonyls, thiobarbituric acid reactive substances (TBARSs)

**Table 2:** Endothelial dysfunction biomarkers.

Vascular adhesion molecule (VCAM-1)	Endothelial progenitor cells (EPCs)
C-reactive protein (CRP)	Circulating endothelial cells (CECs)
Interleukin (IL18)	Osteopontin (OPN)
Fibrinogen	Endothelin
Reactive oxygen species (ROS)	Ischemia-modified albumin (IMA)
Tissue plasminogen activator (tPA)	Cardiac troponin T
Free fatty acids (FFA)	Copeptin
Vascular endothelial growth factor (VEGF)	Platelet-derived growth factor (PDGF)

**Table 3:** Markers of oxidative stress and antioxidants.

Markers of Oxidative Stress	Antioxidants
Lipid peroxidation	Enzymatic
Malondialdehyde (MDA)	Superoxide dismutase (SOD)
Thiobarbituric acid reactive substances (TBARSs)	Catalase
4-hydroxynonenal (HNE)	Glutathione peroxidase (GSH-Px)
Protein oxidation	Non-enzymatic
Protein carbonyls	Glutathione (GSH)
Nucleic acid oxidation	Vitamins (C and E)
8-hydroxydeoxyguanosine (8-OHdG)	

## Data Availability

The data presented in this study are available from the corresponding author upon reasonable request.
